# Shaping the regulation of the *p53* mRNA tumour suppressor: the co-evolution of genetic signatures

**DOI:** 10.1186/s12885-019-6118-y

**Published:** 2019-09-13

**Authors:** Konstantinos Karakostis, Robin Fåhraeus

**Affiliations:** 1 Université Paris 7, INSERM UMR 1131, 27 Rue Juliette Dodu, 75010 Paris, France; 2 Department of Medical Biosciences, Umea University, SE-90185 Umea, Sweden; 3 RECAMO, Masaryk Memorial Cancer Institute, Zluty kopec 7, 656 53 Brno, Czech Republic

**Keywords:** RNA world, *Ciona intestinalis*, Transcription factor, Intrinsically disordered proteins, Protein-RNA interactions, mRNA translation, Molecular basis of disease

## Abstract

Structured RNA regulatory motifs exist from the prebiotic stages of the RNA world to the more complex eukaryotic systems. In cases where a functional RNA structure is within the coding sequence a selective pressure drives a parallel co-evolution of the RNA structure and the encoded peptide domain. The p53-MDM2 axis, describing the interactions between the p53 tumor suppressor and the MDM2 E3 ubiquitin ligase, serves as particularly useful model revealing how secondary RNA structures have co-evolved along with corresponding interacting protein motifs, thus having an impact on protein – RNA and protein – protein interactions; and how such structures developed signal-dependent regulation in mammalian systems. The p53(BOX-I) RNA sequence binds the C-terminus of MDM2 and controls p53 synthesis while the encoded peptide domain binds MDM2 and controls p53 degradation. The BOX-I peptide domain is also located within p53 transcription activation domain. The folding of the *p53* mRNA structure has evolved from temperature-regulated in pre-vertebrates to an ATM kinase signal-dependent pathway in mammalian cells. The protein – protein interaction evolved in vertebrates and became regulated by the same signaling pathway. At the same time the protein - RNA and protein - protein interactions evolved, the p53 trans-activation domain progressed to become integrated into a range of cellular pathways. We discuss how a single synonymous mutation in the BOX-1, the p53(L22 L), observed in a chronic lymphocyte leukaemia patient, prevents the activation of p53 following DNA damage. The concepts analysed and discussed in this review may serve as a conceptual mechanistic paradigm of the co-evolution and function of molecules having roles in cellular regulation, or the aetiology of genetic diseases and how synonymous mutations can affect the encoded protein.

## Background

The p53 tumor suppressor and its main regulator, the MDM2 E3 ubiquitin ligase, constitute a fine model to understand molecular co-evolution, conservation and adaptability, as well as the molecular basis of cancer or other genetic diseases [1–5]. It was recently shown that the BOX-I motif of the transactivation domain of p53 has co-evolved with its regulator MDM2, both at the RNA and the protein levels, leading to the evolution of an intimate regulatory script adopting distinct roles in various stress-induced signalling pathways [4]. Under normal conditions, MDM2 promotes the degradation of p53 via protein-protein interactions while following genotoxic stress, the stress sensor ATM kinase is activated by double-stranded DNA breaks and phosphorylates MDM2(S395) inducing a conformational change which dramatically increases the affinity of MDM2 for the *p53* mRNA [6–14]. The stress-induced MDM2-p53, protein-RNA interaction leads to the stabilisation of p53 via a mechanism whereby MDM2 becomes a positive regulator of p53 [10, 15, 16]. The p53-MDM2 axis contributes a few very important implications and may serve as a paradigm, both mechanistically and conceptually, to understand mechanisms of cellular signalling, the role of intrinsically disordered domains, the role of molecular signatures and interacting motifs as well as their co-evolution, deriving from selective pressure.

This review further highlights novel discoveries on functional interactions between molecular partners, both at the protein - protein and the protein - RNA interaction levels and how p53 evolved from an ancient p53/p63/p73 protein having roles in development, to become a tumor suppressor with numerous interacting partners and functions [17–20]. Findings from in vitro studies on co-evolutionary structural modifications on the interacting motifs and the stereochemically flanking domains on p53 and MDM2 regulating the expression and stabilisation both at the RNA and protein levels from pre-vertebrates, are presented and discussed. These results are set into context with previous evidences supporting a model whereby RNA structures interacting with peptidic motifs may have co-evolved from early prebiotic environments of the RNA world hypothesis to adopt an intimate biochemical relationship with various molecular and cellular functions. The concepts discussed here thus give insights on the nature of the guiding force of the evolution and on a strategy to identify molecular profiling signatures within key players regulating the cellular processes or the development of genetic diseases.

## Main text

### Transition from an RNA world

Life-forms require at least three biopolymers (DNA, RNA and proteins) that mediate the biochemical processes of DNA replication, transcription and RNA translation. This well-orchestrated complex machinery strongly implies its evolution from a more simple system [21]. Recently, a chemical reaction network building up 9 of the 11 intermediates of the biological Krebs (or tricarboxylic acid) cycle, was observed. Such chemical reactions may represent prebiotic precursors to core metabolic pathways [22]. The RNA World hypothesis, describing an intermediate stage of life [23] is a proposed model of ancient biochemistry where structured RNA acquires catalytic properties [24, 25]. Close geological settings and environmental conditions undergoing specific changes (e.g. in the pH) and mixtures of simple chemical compounds could form the required precursors for the prebiotic RNA synthesis. Indeed, a mixture of hydrogen cyanide (HCN) and hydrogen sulphide (H2S) activated by ultraviolet light was shown to effectively form the required precursors of nucleotides, amino acids and lipids [26, 27]. The first polynucleotides are suggested to be small oligomers formed randomly or by non-enzymatic template-copying, via such conditions that promote a feedback between molecular activity and fitness, whereby certain sequences gained a competitive advantage. Chemical properties such as the charge and the hydrophobicity [21, 28] and an early achieved biopolymeric chirality are suggested to have adequately promoted the selection of certain RNA sequences from a vast heterogenous pool of chemical precursors which may catalyse the formation of amide bonds [29, 30] and enforce an enantiomeric selection of peptidic products [31].

As such, early metabolic processes which evolved with a selective preference for certain amino acids, associated with cognate adapter RNAs (tRNA) and their pairing to a linear genetic molecule (mRNA) leading to ordered peptide synthesis (ribosome), is possible [32]. A selection of higher activity sequences can be enforced within lipid vesicle compartments, resembling to encapsulated cell-like systems forming nucleocapsids and protein assemblies that acquire virus-like genome packaging and protection [33, 34]. Such assemblies are currently developed for biomedical applications generating artificial protocells and translational regulatory systems [35]. The transition from an RNA world to self-replicating systems employing DNA, could explain how DNA genomes have evolved via molecular interactions leading to increased biological complexity [36, 37] and to the formation of an early RNA enzyme (ribozyme) capable of copying RNA molecules [23, 38, 39] or even assembled DNA genomes [40]. Additionally, it was recently shown that 3D structural motifs promoting viroid RNA folding, constitute a critical constraint in the RNA–RNA and RNA–protein interactions regulating the viroid genome evolution. Accordingly, only mutations which did not disrupt the structure and function were retained in the population [41]. Such examples illustrate how specific secondary RNA structures and motifs may resemble to nucleation points acting as molecular scaffolds on which interfaces co-evolve along with cognate sites on partnering molecules, to expand the functional cues of their interplay.

In conclusion, the transition form a system as described in the RNA world hypothesis, consisting of small peptides and small RNAs encapsulated in enclosed settings, could be mediated via a co-evolutionary simultaneous lengthening of precursor molecules which selectively reinforced the formation and survival of structured co-evolving motifs [42].

### Shaping the evolutionary path: the interplay between RNA and protein

It has become evident that secondary RNA structures and specific motifs play a crucial role in the interaction with proteins [43] and the underlying mechanisms of protein–RNA interaction have started to unravel [44, 45]. Mutations taking place on structurally sensitive sites on the mRNA that have a disruptive effect on the secondary structure cause aberrant gene expression and their adaptation depends on the expression levels [46]. A recent study, has presented a comprehensive resource database for synonymous mutations from human cancers, emphasising how such single point mutations may have an impact on the expression as well as on the mRNA secondary structure, splicing, RNA stability, RNA folding, and co-translational protein folding [47]. It becomes clear that synonymous mutations affecting certain mRNA secondary structures, may have a strong impact on vital cellular processes. Indeed, the structure of an RNA may favor the accumulation of genetic variation in proteins [48] or regulate the efficiency of translation via several mechanisms. Examples include the 5′ UTR–mediated initiation and stabilization [49], the group of riboswitches [50] and the double-stranded RNA-activated protein kinase [51]. RNA structural features also modulate the dynamics of protein folding during protein synthesis [52]. Specific RNA motifs have been proposed to drive the high mutation rate and the genetic viral variation leading to the formation of quasispecies [53] in the human immunodeficiency virus 1 (HIV-1) [52, 54] as a result of a reciprocal interference between selection at the RNA and protein levels. A fine example of RNA adaptation in a co-evolutionary setting is how the HIV-1 uses the APOBEC3-Vif interaction (host) to modulate its own mutation rate in harsh or variable environments [55]. In eukaryotes, the biological specificity of RNA binding proteins (RBPs) for RNA is affected by the RNA structure and its concentration [56, 57]. Indeed, post-transcriptional gene regulation and RNA processing mediated by proteins adapted to splice introns (RNA splicing) results from their co-evolution with partner RNAs [58]. RBPs play critical roles in post-transcriptional regulation of gene expression in all domains of life [59], having roles in development, cell cycle and signalling mechanisms.

Proteins with RNA-related functions constitute the 7.5% of the human proteome (a set of 1542 proteins) [60, 61] and the occurrence of intrinsically disordered regions (IDRs) within RBPs appears to be conserved and expanded from yeast to humans, often in the form of repeats that co-evolved with the increasing complexity of eukaryotic transcriptomes [62]. IDRs are abundant in RBPs and approximately 20% of mammalian RBPs contain disordered regions which correspond to over 80% of their complete structures/sequences [63]. IDRs are subjected to strong sequence constraints [61], implying crucial functional roles in the intermolecular and intramolecular interactions. A fine example is the RBP-dependent regulation of the p53 tumor suppressor, whereby RBPs, such as RBM38, interact with the 5′ or 3′ UTR of *p53* mRNA thus playing pivotal roles in both maintaining genomic integrity and tumor suppression [64]. The p53 itself comprises IDRs dramatically increasing the potential of the p53 interactome [65] and an extensive description of *p53* mRNA interacting factors in context of various cellular conditions and stress responses, was presented recently [66]. A computational model describing how proteins associate with extended RNA elements may aid the discrimination between regulated and non-regulated RNAs and reveal potential regulatory motifs, by giving insights on the consequences of mutational events towards the binding activity [67]. In another computational approach, about 80 cellular processes that can be regulated at the post-transcriptional level were identified by analysis of protein–mRNA interaction networks from more than 800 human RBPs; and mechanistic regulatory hypotheses were proposed [68]. These studies show that there is growing interest in identifying cellular roles of RNA - protein interfaces in the context of human diseases and it would be of great interest and usefulness to include the well-studied *p53* mRNA - MDM2 axis.

Other factors influencing the RNA-protein interplay thus shaping their co-evolutionary path by balancing the intrinsic conflict between stability and aggregation, include the chaperone Hsp90 [69]. Hsp90 modulates trade-offs among protein stability and increased aggregation and hydrophobicity, acting both at the polypeptidic level and the RNA level. Acting as a chaperone, it reduces the aggregation of intermediate folding states of proteins while regulating the translation speed, thus leading to more stable and versatile proteins. In the eukaryotic cell, Hsp90 promotes evolutionary changes in otherwise entrenched developmental processes, by functioning as a buffer allowing the accumulation of silent sequence variants under neutral conditions, which become selected upon conditions while Hsp90 activity is compromised. In this way, certain variants gain the prospect to get enriched by selection and even rapidly become independent of the Hsp90 mutation [70].

Additionally, it has been shown that post-translational modifications (PTMs) are known to regulate RNA-binding proteins [71] as well as proteins that are regulated via complex mechanisms employing RNA. It has indeed become evident that the interaction of secondary RNA structures with respective RBDs in proteins is favoured in sequences including IDRs and in substrates where modifications take place. These factors, along with the activity of chaperones and of post-transcriptional and post-translational mechanisms, regulate the morphological co-evolution of such RNA-protein partners. A particularly interesting and useful example is the interaction of the p53 tumor suppressor with MDM2, which is employed in this review as a model to describe the co-evolution of protein-mRNA structures with functional outcomes; and it is discussed in detail in the following sections.

### Τhe evolution of the p53 family from an ancestral molecule

**Fig. 1:**
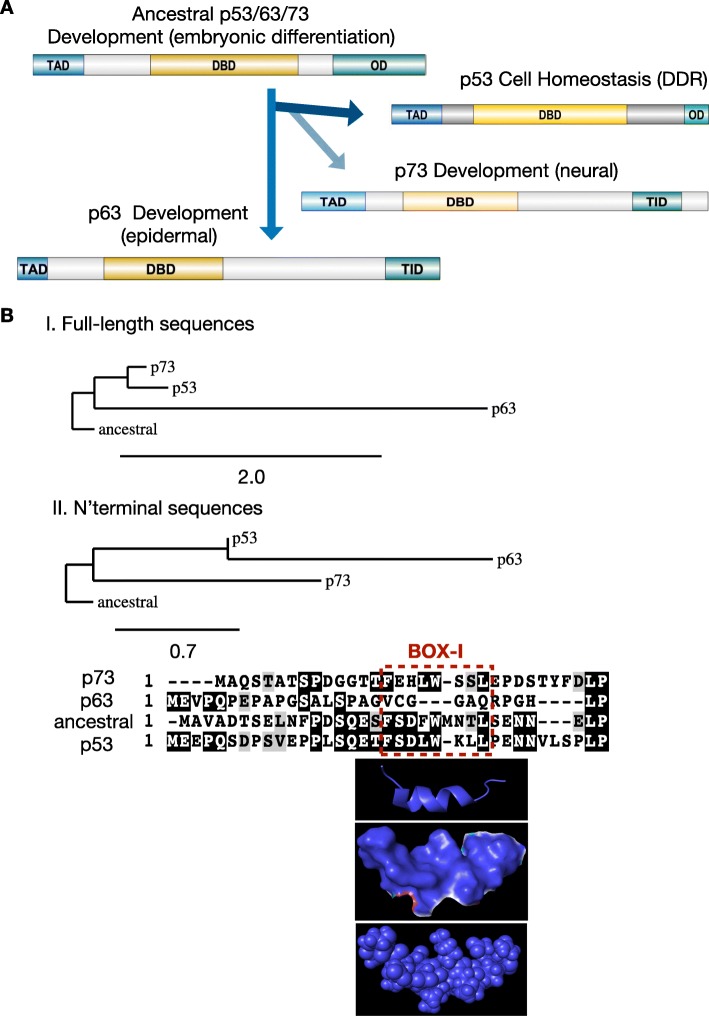
The evolution of p53. **a** Illustration of the roles and the main functional protein domains of proteins of the p53 family. Ancestral p53/p63/p73 has roles in development and embryonic differentiation, giving rise to three genes with distinct roles on cellular homeostasis and cancer (p53) and on epidermal (p63) and neural (p73) development. TAD: transactivation domain; DBD: DNA-binding domain; OD: oligomerization doamain; TID: transcription inhibition domain. The illustration of the domains has been prepared with the software DOG for the visualisation of protein domain structures. **b** Phylogenetic tree comparing the ancestral p53/p63/p73 protein from the pre-vertebrate *Ciona intestinalis* with the human proteins p53, p63 and p73. Both the full-length (I) and the partial N-terminal sequences (II) of the ancestral p53/p63/p73 protein show higher overall homology to p73 and p53 as compared to p63. The sequences were retrieved by the NCBI database: ancestral p53/63/73: (NP_001071796.1); p53 (BAC16799.1); p63 (AAB21139.1) and p73 (AAC61887.1). The aligned N-terminal sequences used in this analysis are the following: ancestral p53/63/73: (NP_001071796.1, aa 1–33); p53: (BAC16799.1, aa 1–36); p73: (AAC61887.1, aa 1–32); and p63: (AAB21139.1, aa 1–30). The alignments and the trees were prepared by the software found on phylogeny.fr [76]. The phylogenetic distances values are noted. The pdb file 2MWY was used for the modelling of the extended region SQETFSDLWLLPEN of p53, including the BOX-I motif FSDLWLL

In mammals, the p53 family consists of the p53, p63, and p73 proteins. P63 and p73 have important roles in development [72, 73]. While the p63 protein has a role in skin and epithelial development, p73 has an important role in neuronal development and differentiation [17]. Activated by cellular stresses such as genotoxic stress [8, 74] and endoplasmic reticulum stress [75], p53 has evolved to play a key role in cellular homeostasis as a tumor suppressor and as a signal response factor preventing oncogenesis (Fig. 1, a).

The proteins of the p53 family have evolved from whole genome duplications of a single ancestral p53/p63/p73 protein in the vertebrates while invertebrate p53 superfamily members appear to have a p63-like domain structure [77, 78]. Evolutionary studies have identified various homologues of the ancestral protein in the placozoan *Trichoplax adhaerens* [1], in Nematoda roundworms (Caenorhabditis) [79], in the pre-vertebrate *Ciona intestinalis* [4], in the invertebrate *Mytilus trossulus* [80], in the early vertebrate cartilaginous fish, as well as in Cnidaria like the starlet sea anemone (*Nematostella vectensis*), and in flies (Drosophila) [79]. The phylogenetic relationships of metazoan genes containing p53/p63/p73 transactivation domains (TADs) and corresponding p53/p63/p73BD on MDM2, in context of their co-evolution, have been thoroughly investigated and structural models comparing the FxxxWxxL motifs (box-I) were presented [5]. A detailed phylogenetic analysis of the evolution of the p53 family with a particular focus on the TAD and its co-evolution with the binding domain on MDM2 showed that these sequences are significantly conserved from early metazoan time [5]. Such predictive and experimental studies focusing on the interfaces of structured RNA and protein binding epitopes, are highly encouraged. The function of this ancestral gene in the early metazoan sea anemone was suggested to be related with the protection of the germ-line gametes from DNA damage while the first functional change coincided with the development of stem cells and progenitor cells, designed to regenerate somatic tissues over the life time of adult organisms. As with germ cells, this has allowed the possibility of unlimited cell growth and the development of cancer [79]. However, the function of the ancestral p53/p63/p73 and how it later evolved into three functionally diverse proteins that share a certain homology, has only recently started to unravel. Phylogenetic analyses have shown that the p53/p63/p73 family has been duplicated multiple times during the evolution, from the invertebrates [81]. In the vertebrate lineage leading to fishes, reptiles and mammals, duplications gave rise to three distinct genes (p53, p63 and p73) that are retained in the majority of species [5]. Such p53 gene duplications are suggested to contribute to an enhanced induction of apoptosis via a hyperactive TP53 signaling pathway activated in response to DNA damage and they have been linked with the evolution of large body sizes and with the resolution of Peto′s paradox that correlates the body size with the risk of cancer [2, 82]. A phylogenetic analysis of the evolution of the ancestral p53/p63/p73 protein from the pre-vertebrate *Ciona intestinalis* and the p53, p63 and p73 proteins from human, shows that the full-length sequence of the ancestral protein is more closely related to human p53 and p73, as compared to p63 (Fig. 1**,** b I). Similarly, the aligned sequence of the N-terminal within the ancestral transactivation domain (TAD) (aa 1–33) shows a higher similarity with the corresponding sequences on p53 (aa 1–36) and p73 (aa 1–32) as compared to p63 (aa 1–30) (Fig. 1**,** b II). During evolution, a distinguished accelerated expansion of various gene families coding for transcription factors has been observed and it is often predated by modular domain rearrangements, forming new sub-families in terms of protein–protein interactions. This separation allows for radical shifts in the functional spectrum of duplicated transcription factors [83]. Such domain rearrangements can take place on modular regions involved in molecular binding and the modular organization of binding sites is shown to confer robustness and functional flexibility, facilitating the evolution of protein interactions [84]. Gene evolution at the domain level has been an interesting point of study [85]. An example of modular evolution is the BOX-I motif of p53, analysed here.

### A key evolutionary signature: the BOX-I motif of the transactivation domain of p53

All members of the mammalian p53 family are transcription factors containing an N-terminal transactivation domain (TAD), a DNA-binding domain and a C-terminal oligomerization domain. Even though they exhibit a high overall sequence and structural similarity, their N-terminal TADs are not well conserved [4, 86, 87]. The TAD sequence of the mammalian p53 is well-conserved and forms an amphipathic a-helix domain (aa. 19–26, BOX-I) [7, 88, 89] which exhibits steric complementarity with the N-terminal hydrophobic cleft (aa 26–108) of the E3 ubiquitin ligase MDM2 oncoprotein. The TAD domain of p53 confers the p53 transcriptional transactivation in mammals and is thus under selective pressure in the evolution. The p53 transactivation activity is mediated via two adjacent but functionally specialized domains (TAD1 and TAD2), transactivating different target genes and effector pathways [90, 91]. Yet, TAD1 plays a predominant role over TAD2, and is required for DNA damage-induced G1 arrest and apoptosis, but not for RAS-induced senescence in fibroblasts, explaining the evolutionary pressure to split the transactivation function into two domains with different panels of co-factors and modifying complexes that confer a more robust and dynamic (context-dependent) transactivation activity [92]*.* The BOX-I motif of the TAD1 of p53 (aa. 19–26) is the most conserved region of p53 and was shown to play a dual role on its interaction and regulation of p53 by MDM2; one at the RNA level and one at the protein level [14, 93]. These interactions evolved from the pre-vertebrate ancestral p53(ΒΟΧ-Ι) as a result of co-evolution with the MDM2 protein [4]. In other studies structural models comparing FxxxWxxL peptidic motifs (BOX-I) have also shown that the p53(BOX-I) - MDM2 interplay dates back to early metazoan time [5]. However, to our knowledge, there are no additional studies focusing on the molecular evolution of the *p53(box-I)* mRNA structure and on the interactions with MDM2 homologues from various species. Such experimental and predictive studies with an emphasis on the functional roles of such molecular interfaces on co-evolved molecular partners, are highly encouraged and anticipated.

### The co-evolution of p53 and MDM2 proteins

The TAD domain of p53 comprises the co-evolved epitopes mediating the p53 - MDM2 interaction. P53 is tightly regulated by MDM2. Under normal conditions, MDM2 negatively regulates p53 by promoting p53 ubiquitination and degradation via the 26S proteasomal pathway and by blocking its TA activity. However, during genotoxic stress, MDM2 switches to become a positive regulator of p53. The underlying mechanism also involves MDMX (HDMX), a close homolog to MDM2 which has no E3 ubiquitin ligase activity [93, 94]. Phosphorylation at serine MDM2(S395) and MDMX(S403) by the ATM kinase induces conformational changes both on MDM2 and MDMX [94, 95], allowing their RING domains to bind the *p53* mRNA sequence that encodes the BOX-I motif [93], resulting to an increase in p53’s rate of synthesis and to the suppression of the MDM2’s E3 ubiquitin ligase activity [10, 89, 93, 96]. Hence, two MDM2-interacting motifs with opposing functions towards p53 expression have evolved from the same genomic sequence of p53: one at the mRNA level and one peptidic [4, 15] and these peptide- and RNA- motifs interacting with MDM2 are encoded by the same conserved BOX-I sequence. In an attempt to describe how this molecular co-evolution took place, it has been shown that the evolution of the p53 peptide- and RNA- interactions with MDM2 have been influenced and selected by different cues from pre-vertebrates to vertebrates [4]. Short engineered peptides of the *C. intestinalis* or mammalian BOX-I sequences bind similarly well to both either *C. intestinalis* or mammalian MDM2 proteins. However, when the full length p53 proteins were tested, Ci-p53wt showed an interaction with either Hu-MDM2 and Ci-MDM2 protein, but the Hu-p53wt showed poor affinity for Ci-MDM2 in ELISA experiments [4]. These results strongly indicated that the allosteric interference imposed by the C-terminal flanking region of the BOX-I domain prevents the p53-MDM2 protein–protein interaction. Indeed, it has been experimentally shown in the pre-vertebrate *C. intestinalis* that the region encompassing the residues Q41 to F56, prevents the interaction and deletion of these flanking residues (Q41 to F56) not only restored the interaction between *C. intestinalis* p53 protein and MDM2 but it also induced the binding to the DO-I mAB which binds the BOX-I domain [4]. This was confirmed in Proximity Ligation Assay (PLA) experiments [4]. The PLA microscopy is a *state-of-the-art* microscopy-based technique that provides an in situ semi-quantitative estimation of endogenous molecular interactions/associations that occur in a low frequency and/or mediated by molecules of a low concentration; and are thus considered to be of a low abundance, in comparison to numerous other interactions taking place in the cell [97–99]. It offers unique information on the sub-cellular localisation of molecular interactions between proteins or between RNA and protein. These findings strongly indicated that p53 has evolved from an ancestral p53/p63/p73 gene to interact with MDM2 by elimination of the encoding flanking region of BOX-I which paved the way for MDM2 to take a negative regulatory role on p53. These findings highlighted that computational studies and molecular evolutionary models based on sequence alignment, should also take into account 3D structural evidences or models. In line with this notion, an evolutionary study on a molecular level of the p53/p63/p73 TAD domain with regard to protein disorder and regulatory properties, showed similarities in the phosphorylation pattern of vertebrate p53 and mollusk and annelid p53/p63/p73, implying that functional properties of regulation via phosphorylation were already present in p53/p63/p73 from deuterostomes (e.g. Chordata) and protostomes (e.g. Mollusca and Arthropoda) [5]. Additionally, a phylogenetic analysis on the p53/p63/p73 and MDM proteins from phyla that retain the interaction domains TAD and p53/p63/p73 BD (binding domain), based on both vertebrate and invertebrate species showed that the signaling pathway of the TAD and p53/p63/p73BD has co-evolved or disappeared in distinct lineages [5]. In line with this, evolutionary studies have suggested that the MDM2 – p53 interaction is present in early metazoans and was later lost in some species, but it is not clear whether this event occurred in the pre-vertebrates or earlier in the evolution [1, 5].

In conclusion, alternations in the intrinsically disordered p53 N-terminal sequence of pre-vertebrates shaped the conformation and the presentation of the conserved BOX-I peptide motif (TAD) to interact with MDM2 in vertebrates, allowing MDM2 to become a negative regulator of p53.

### The co-evolution of the *p53* mRNA - MDM2 protein interaction

**Fig. 2:**
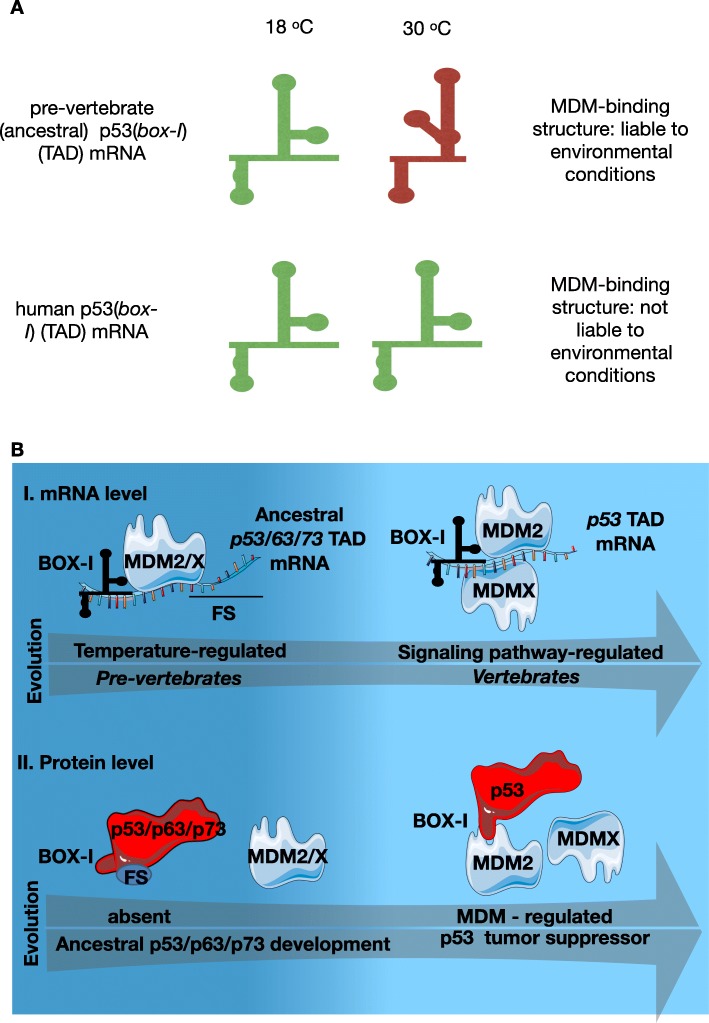
Cartoon illustration of the evolution of the *p53* mRNA structure and the co-evolution of p53 and MDM2. **a** Illustration of the structures of *p53(box-I)* in the transactivation domains (TAD) of mRNA sequences from the pre-vertebrate (ancestral) and human p53. The structure and the MDM2-interaction of the ancestral *p53* mRNA is temperature dependent and its binding to the ancestral MDM2/X is optimal at 18 °C (temperature of the natural environment of *C.intestinalis*) while the human homologues (MDM2 via MDMX) positively interact on either 18 °C and 30 °C temperatures. **b** Co-evolutionary pressure of the box-I motif of the transactivation (TA) domain of p53 and the p53 binding site on MDM family, at the RNA and protein levels. (i) RNA level: Ancestral MDM2/X interacts with the p53 mRNA in a temperature- dependent fashion while the in MDM2 requires the employment of MDMX and the activity of a p53 signaling pathway, responding to DNA damage response. (ii) Protein level: The p53 - MDM2 protein - protein interaction has evolved by changes in the flanking sequence (FS) of box-I and it is intimately related to the evolution of the p53 activity as a tumor suppressor

In mammals the *p53* mRNA - MDM2 protein interaction is facilitated via MDMX [12, 100] which is found in mammals but not in the pre-vertebrate *C.intestinalis.* Following phosphorylation by the ATM kinase during the DNA damage response, MDMX binds the nascent *p53* mRNA forming an RNA platform/structure on which MDM2 can bind and stimulate p53 synthesis. This mRNA structure is found on the 5′ of the *p53* mRNA coding sequence (within the first 120 nt) and consists of three stem loops [12, 101]. In the pre-vertebrates the catalytic role of MDMX for the *p53* mRNA - MDM2 interaction is influenced by temperature. Temperature was shown to govern the folding of the ancestral *Ci-p53* mRNA and its binding to Ci-MDM2, by inducing a stem-loop structure within the *box-I* RNA motif. This structure interestingly resembles to the mammalian p53 homolog that has a high affinity for MDMX and MDM2, following DNA damage [4, 12, 15], (Fig. 2**,** a), indicating a putative role of the ancestral MDM2 as a positive regulator of p53. The RNA structures were solved experimentally by the in vivo DMS Footprint Assay and the in vitro DMS modification assay [4] that use dimethyl sulphate (DMS)-modified RNA and sequencing to reveal the RNA structure. The box-I-coding Ci-*p53* mRNA sequence adopts a temperature-dependent structure that governs the interaction with Ci-MDM2. This interaction was experimentally confirmed by RNA co-immunoprecipitation coupled with qPCR and ELISA and it was visualized on fixed embryos using a modified version of the PLA, the RNA PLA [4, 10, 102]. Similar findings of temperature-dependent structural adaptations of transcripts were identified in Protozoa, Nematoda, Cnidaria, and Tunicata [103] while temperature was shown to influence mRNA structures and translation in various organisms [104–106], including bacteria [107], yeast [108]; corals [109], and plants [110]. It has been particularly interesting to track in the evolution how the temperature-dependent structured pre-vertebrate *p53(box-I)* mRNA becomes folded in an MDMX–dependent fashion in vertebrates and mammals while at the same time the encoded BOX-I motif and its flanking region within the encoded TAD, evolved to facilitate the protein - protein interaction [4] (Fig. 2**,** b).

In conclusion, the steric complementarity of the MDM2 - *p53* mRNA interaction requires a specific *p53(box-I)* mRNA structure, which has evolved within the *p53* mRNA sequence. This temperature-induced RNA structure in the pre-vertebrates evolved to become MDM2 and MDMX-dependent, indicating a putative role of the ancestral MDM2 as a positive regulator of p53.

### The regulation of p53 by MDM2 at the protein and RNA levels

The p53 tumor suppressor and the p53 regulatory axis is a fine model constituting a complex ATM kinase - dependent regulation system [10, 14, 111, 112] that involves: (1) the employment of post-transcriptional regulation by RBPs [64]; (2) the employment of IDRs conferring multi-functionality [65]; (3) the employment of post-translational modifications following different stress responses and (4) the formation of a multifactorial mechanism, employing various additional proteins, as it has evolved from a more simple system [4, 14]. Recent findings showing the functional consequences of a single synonymous cancer mutation on the *p53* mRNA (L22 L) that abrogates the interaction of the *p53* mRNA with the MDM2 E3 ubiquitin ligase protein [14], highlight the notion that certain structured RNA motifs constitute a signature of regulation and urge the attention for more detailed studies on similar molecular partners and networks. One of the key points of this review is to analyse and discuss the role of the *p53* mRNA in the regulation of p53 and to give insights on the notion that certain structured motifs, such as the *box-I* motif of p53, may be encoded by sequences which are prone to adaptive mutation (sequence-dependent ‘hotspot’ [113]) thus constituting co-evolved structures allowing molecular interactions between RNA and protein.

**Fig. 3:**
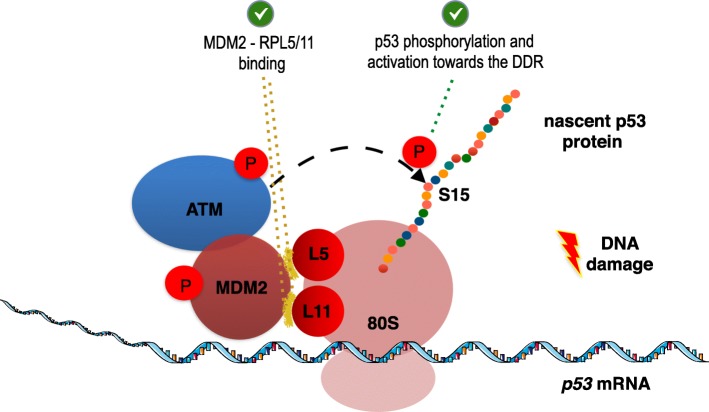
Model of the p53 translating ribosome formed by MDM2, following DNA damage. The interactions of MDM2 with the ribosomal proteins RPL5 and RPL11 (highlighted in yellow colour), are required for the phosphorylation of the nascent p53 peptide by ATM, leading to its stabilisation and activation towards the DNA damage response

The conservation of the mammalian MDM2 - p53(box-I), protein-protein and protein-RNA interactions has become evident and their functional consequences have started to unravel. Under normal conditions MDM2 acts as a negative regulator of p53. MDM2 binds the p53(BOX-I) protein and targets it for degradation via the 26S proteasomal pathway. However, following DNA damage, the ATM kinase is autophosphorylated and phosphorylates p53(S15) preventing the p53-MDM2 protein-protein interaction. Additionally, ATM phosphorylates MDM2(S395) which in turn develops an affinity for the *p53* mRNA. This event also leads to the prevention of the binding of MDM2 on the p53 protein. These phosphorylation events lead to the stabilisation of p53 [3, 114–117]. Thus, there are at least two phosphorylation events leading to an MDM2-dependent regulation of p53: (a) phosphorylation of p53(S15) abrogating the binding of MDM2 and preventing the ubiquitination and the proteasomal targeting of p53; and (b) phosphorylation of MDM2(S395) promoting the interaction of MDM2 with the *p53* mRNA thus leading to enhanced p53 synthesis. A recent study experimentally showed how these events are orchestrated via a mechanism whereby phosphorylated MDM2(S395) acts as a *p53* mRNA-dependent carrier that mediates the formation of a complex at the cytoplasm. This complex, consists of the MDM2, the *p53* mRNA, the ribosomal proteins RPL5 and RPL11 and the ATM kinase and was shown to promote the synthesis and activation of p53 protein while preventing its degradation [14]. To analyse these interactions, the p53(L22 L) synonymous mutation was used. L22 L at the codon in position 22, CUA to CUG, is located in the apical loop of the hairpin U180-A218, and was initially observed in a chronic lymphocyte leukaemia patient [101, 118]. The p53(L22 L) synonymous mutation is located at the TAD domain of p53 which constitutes the MDM2 binding site and it prevents the interaction of the *p53* mRNA with MDM2 by disrupting the p53 hairpin-MDM2 interaction [15]. It was also shown to impair p53 and Δ40p53 synthesis [118, 119] and to lead to a poor stabilization of the encoded p53 protein following genotoxic stress [14, 15]. P53(L22 L) showed poor affinity for MDM2 and this resulted to a poor MDM2-dependent positive regulation effect towards p53, following genotoxic stress. Additionally, the MDM2(C305F) and MDM2(C308Y) mutants that prevent the interactions with the ribosomal proteins RPL5 and RPL11 respectively, failed to stimulate the over-expression of p53 following DNA damage. These mutants remained localised in the nucleolus and did not reach the cytoplasm to form the ribosome including the RPL5, RPL11 and the *p53* mRNA (as shown by immunofluorescence). The work of Karakostis et al, [14] presents direct evidences showing that the interactions of MDM2 with (a) the *p53* mRNA and with (b) the ribosomal proteins RPL5 and RPL11 are required for the translocation of the complex to the cytoplasm, supporting the concept that MDM2 acts as a carrier of RP proteins and of the *p53* mRNA at the cytoplasm which promotes the formation of the ribosome that translates the *p53* mRNA (Fig. 3).

Consequently and in parallel with the release of MDM2 from the translating *p53* mRNA, the under-synthesis nascent p53 peptide is phosphorylated on S15 by the ATM kinase which has hitch-hiked at the p53-translating ribosome by MDM2 and it is thus localised in close proximity with the nascent p53 peptide. This phosphorylation prevents the interaction of the newly synthesized p53 peptide with free (not binding the *p53* mRNA) MDM2, leading to its stabilization. Evidently, as p53 synthesis continues, MDM2 will be displaced from the translating *p53* mRNA. In human, the binding platform consists of three stem loops within the first 120 nt of the encoding sequence of the *p53* mRNA. Due stereochemical constrains, ATM may bind the nascent p53 protein and phosphorylate S15 only at the point when about 45 aa have been synthesised. This is qualitatively consistent with MDM2 becoming displaced from the *p53* mRNA. In parallel, MDM2 interacts with RPL5 and RPL11 as well with other ribosomal proteins, such as the RPL37, RPS15 and RPS20 independently of p53, as it has been shown in H1299 cells (p53 null) [120, 121]. Conclusively, MDM2 remains bound on the translating ribosome until p53 is synthesised and phosphorylated by ATM at S15. This was further confirmed by PLA and PLEA on purified polysomes [14]. The Proximity Ligation ELISA (PLEA) is a novel assay that combines the PLA with the ELISA in order to obtain quantitative affinity values of the interactions under study [14, 122]. PLEA offers the possibility to study the interaction of three molecules, by using three primary antibodies; the capture antibody used to capture the complexed set of proteins on a well plate and a set of two additional antibodies is used for the amplification of the PLA signal [14]. As a result, this model describes how ATM targets the nascent p53 peptide and phosphorylates the S15, thus serving as a rapid mechanism whereby an activated p53 pool is synthesised in response to DNA damage [14]. This comes in line with the notion that post-translational modifications on p53, including phosphorylation, acetylation, ubiquitination, sumoylation, neddylation and methylation may not only promote p53 stabilization but also confer p53 specificity to certain target genes [123], as well as regulate the specificity of post-translational modifications on p53, in response to other stresses, such as oxidative stress [124] or ER stress [125] and such studies are highly anticipated.

As a general conclusion from the described model, mutations on the mRNA can signal post-translational modifications of the encoded protein and may involve a fine-tuning activity by which the stabilization and the activity of the encoded protein towards certain stresses (in this case DNA damage), is decided and regulated at the cognate RNA level. This novel notion of functional aspects of silent mutations has only recently started to unravel [126]. The description of the profound effects of synonymous mutations in the coding region of p53 regulating the interaction between the *p53* mRNA and the MDM2 protein, opens up new processes by which p53 translation is controlled and emphasizes the role of the RNA itself in the regulation of p53 [127]. Additional roles of *p53* mRNA as a target for signalling pathways are listed in a recent review [66] and another example of RNA/protein coevolution comes for the ribosomal protein L22 and neighbouring 23S RNA. This work, based on a theoretic computational method, provided with direct evidence of RNA/protein coevolution driven by biophysical constraints on their interacting RNA and protein chains [128, 129]. It was shown that there is a co-evolutionary pattern (an increased co-evolution likelihood) on residue triplets, and not on duplets, for which mutations in an RNA nucleotide results in a change in residue distribution for proximal amino acids [129].

### Functional consequences of co-evolved molecules

The p53-MDM2 co-evolutionary pattern described here, gives insights on the notion that certain genetically conserved signatures such as the p53(box-I) motif and the corresponding structural domains regulating its presentation to the interacting partner, such as the flanking sequence of the p53(TAD), evolve allosterically and intramolecularly. Extreme interchanging environments limiting the functional barriers while increasing the selective pressure, may favor strict cellular regulation and dedicated molecular networking. As described here, the temperature-regulated ancestral *p53/63/73(TAD)* mRNA structure, managing its interaction with the ancestral MDM2/X in the pre-vertebrate *Ciona intestinalis*, has co-evolved with the MDM family, to become regulated by MDMX in post-vertebrate species. This is an example of co-evolution between interacting and interdependent partners which is favored over the molecular evolution influenced by environmental inducers which may be interchangeable and constantly variable, increasing the risk of lethal phenotypes. The information gained by exploring the sequences of such molecular partners and how they co-evolved to become interdependent at a molecular (structural), cellular (expression, regulation) and functional level, may help in identifying the roles of specific genetic variations and could be considered as a major component of the driving force of the evolution.

In order to gain insights into the functional consequences of transcription factors and targets that modulate the expression of networks to lead the phenotypic diversification among species as well as being implicated in diseases, future studies should focus on mutations occurring both in exons and introns and aim to characterize such variations such as single nucleotide polymorphisms (SNPs) [130]. The transcription factor p53 plays a key role in cancer suppression by preventing the proliferation of cells carrying potentially cancer-prone mutations. This function results from the co-evolution of an ancestral p53/p63/p73 molecule with MDM2 which shaped p53 superfamily to become integrated into a range of cellular pathways and molecular interactions, acquiring hundreds of distinct functions in metabolism, genome integrity, ageing, immune cell response and stress responses; it thus is not surprising that cancer cells require its inactivation. One important point highlighted here is that the co-evolution of p53 with its regulator, MDM2, lies on the evolutionary pressure on the BOX-I and the TA domain, both at the RNA and the protein level (Fig. 2**,** b).

### Conclusive remarks

An MDM2-dependent mechanism leading to the stabilisation of p53 was presented in a model by Karakostis et al*,* [14] describing downstream effects of the *p53* mRNA - MDM2 interaction. The experimental evidences were derived by co-immunoprecipitation, sandwich ELISA and PLA experiments and were further confirmed by PLEA. PLEA confirmed that MDM2 and ATM are co-localised on purified ribosomes, actively translating p53 and that the nascent p53 peptide is phosphorylated on Ser15 epitope by ATM [14]. This is in line with previously described ribonucleoprotein complexes composed of 5S RNA, RPL5 protein, MDM2 proteins, p53 protein [120] and with studies showing the roles of RPL11 and other ribosomal proteins on the regulation of MDM2 [131–134]. MDM2 is also implicated in sensing dysfunctional ribosomal biogenesis leading to the activation of p53 [135–138] and it will be of high interest to test the concepts of this model to study the mechanism whereby the ribosomal precursor complex is formed and how p53 is activated following ribosomal stress. This model explains previous puzzling observations using the artificial silent mutant *p53*^*TriM*^ mRNA, which carries synonymous mutations in codons 17, 18 and 19 and has an increased affinity for MDM2 under normal conditions. p53^TriM^ exhibits a higher rate of MDM2-dependent translation but also a higher rate of MDM2-dependent p53 degradation [14, 15]. Hence, according to the described mechanism, an increase in the MDM2 - *p53* mRNA interaction facilitates the consequent increase in MDM2-dependent p53 synthesis. This is in line with *p53*^*TriM*^ mRNA exhibiting an affinity for MDM2 which is not regulated by the DNA damage response and the ATM phosphorylations, explaining why the nascent p53 in the absence of ATM is efficiently degraded by MDM2 [15]. The observed increased degradation can also be explained by the fact that once the phosphorylated MDM2(S395) is released from the ribosome and from the *p53* mRNA which is being translated, it efficiently ubiquitinates the p53 protein that is not phosphorylated by ATM at S15 [16].

**Fig. 4:**
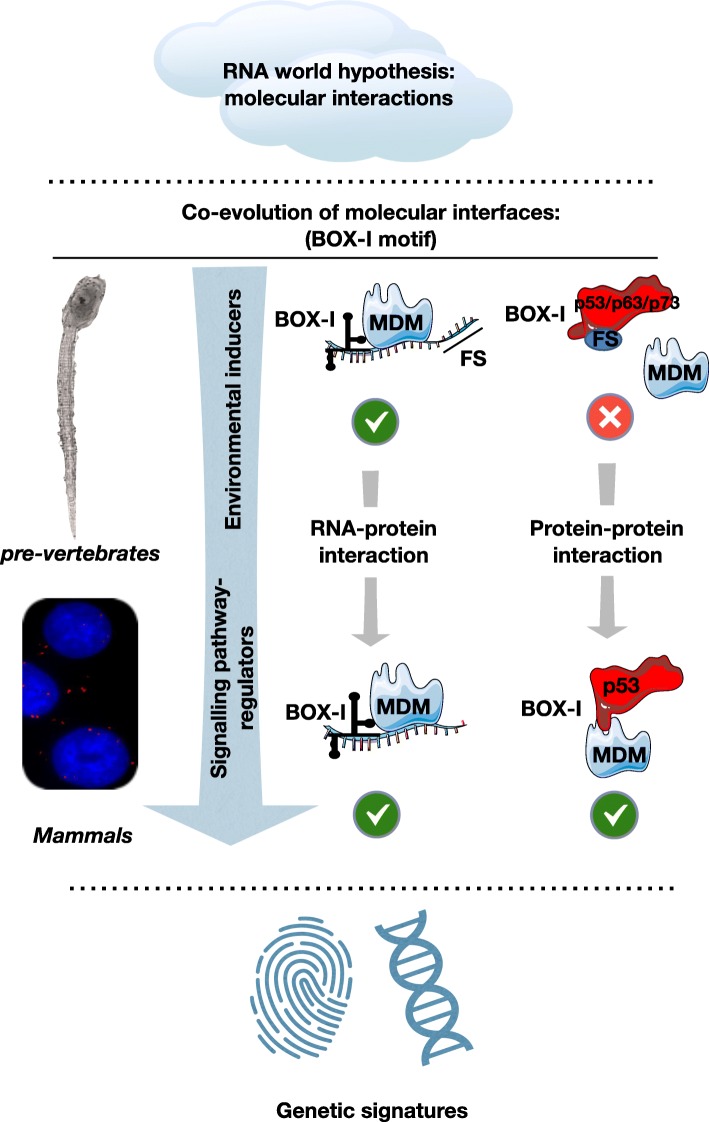
Graphical summary illustrating how molecular signatures, such as the *p53*(*box-I)* motif, co-evolve at the mRNA and protein levels along with interacting partners, such as the MDM2 E3 ligase (MDM family), to mediate the transition from an environmentally-induced type of interaction and function to a well-regulated interplay, driven by specific molecular interactions forming a signalling pathway that mediates the regulation of p53. Such signatures are of high potential value in molecular diagnostics for genetic diseases such as cancer and in precision medicine

Overall, molecular cell biology and evolutionary theories are interlinked with the understanding of mechanisms involved in the development of genetic variations with functional outcomes. Such variations are promoted during the evolution as a result of adaptation, either directly towards environmental factors; or via interactions between co-evolved interacting molecular partners, as described for p53 and MDM2; or even driven by co-evolutionary interactions between species, as described in viruses and bacteria [139–141]. Molecular, biochemical and immunochemical tools for identifying and characterising the interaction of co-evolved variants, are currently being developed, improved and expanded. Techniques such as the DMS footprint for studying RNA structures when combined with the PLA and the PLEA, for studying dynamic molecular interactions of low abundance in situ at the sub-cellular level, open new horizons in studying in vitro and *in cellulo* molecular interactions and establish the possibility to functionally characterise the effect of genetic variations (mutations or polymorphisms) on the molecular interactions; and the co-evolution of the molecular partners. Such functional genetic variations represent key targets for understanding and treating genetic diseases such as cancer [142] as well as for understanding how synonymous mutations, which have largely been ignored, urge for a closer examination [127]. Targeting these genetic variations may be extremely useful in sequencing-based approaches employed for genetic profiling in clinical diagnoses for personalized medicine. Considering variants of co-evolved partners, which may be located both on protein-coding regions (exons) of the genome as well as on intronic and non-coding regions, may be of crucial importance in developing efficient profiling molecular networks. Additionally, it is important for the employed bioinformatic analyses to accurately predict and interpret the effects of such variants involved in gene-expression or RNA splicing [143]. Such studies focusing on synonymous variants and on the structural variations both on the mRNA and the encoded protein motifs, induced either by mutations or via evolutionary cues of co-evolved functional remnants from ancient interactions, may help in answering the question of how cancer shapes evolution and how evolution shapes cancer, and may be extremely useful in developing effective diagnoses and therapies by identifying key genetic signatures involved in genetic diseases (Fig. 4). The p53-MDM2 axis constitutes an ideal model with significant impact on cancer therapeutics [144] that may facilitate the understanding of various systems and interaction networks employing protein-mRNA interactions and refine predictive studies aiming to identify biomarkers of genetic diseases for translation research [47, 68, 145].

## Data Availability

The datasets used and/or analysed during the current study are available from the corresponding author on reasonable request.

## Abbreviations

3D: Three-dimensional
aa: Amino acid
Ci; *C. intestinalis*: *Ciona intestinalis*
DMS: Dimethyl sulphate
ELISA: Enzyme-linked immunosorbent assay
HIV-1: Human immunodeficiency virus 1
IDRs: Intrinsically disordered regions
PLA: Proximity ligation assay
PLEA: Proximity ligation ELISA
PTMs: Post-translational modifications
RBDs: RNA-binding domains
RBPs: RNA-binding proteins
S395: Serine aa 395
Ser15: Serine aa 15
SNP: Single nucleotide polymorphism
TAD: Trans-activation domain
